# Assessing Patients beyond the Simple Optics of BMI: The Concomitant Role of Sarcopenia and BMI in Predicting Kidney Transplant Outcomes

**DOI:** 10.3390/life14081036

**Published:** 2024-08-20

**Authors:** Christopher Seet, Laura Clementoni, Mohammed Rashid Akhtar, Pankaj Chandak, Mohammed Saoud, Amr Elsaadany, Muhammad Magdi Yaqoob, Ismail Heyder Mohamed, Muhammad Arslan Khurram

**Affiliations:** 1 Department of Nephrology and Transplantation, The Royal London Hospital, Bart’s Health NHS Trust, London E1 1FR, UK; 2 Department of Radiology, The Royal London Hospital, Bart’s Health NHS Trust, London E1 1FR, UK; 3 William Harvey Research Institute, Queen Mary University London, London E1 4NS, UK

**Keywords:** kidney transplantation, sarcopenia, obesity, BMI

## Abstract

Background: Body composition is associated with prognosis in many clinical settings, and patients undergoing kidney transplantation are often high risk with multiple comorbidities. We aimed to assess the effect of sarcopenia and body composition on transplant outcomes. Methods: We performed a retrospective analysis of 274 kidney transplants with CT scans within 3 years of transplantation. The skeletal muscle index (SMI) at the L3 vertebrae was used to evaluate sarcopenia (SMI < 40.31 cm^2^/m^2^ in males, <30.88 cm^2^/m^2^ in females). Sarcopenia, body mass index (BMI), and the visceral-to-subcutaneous-fat ratio (VSR) were assessed separately. We also used a composite BMI/sarcopenia measurement in four patient groups: BMI < 25/Non-Sarcopenic, BMI < 25/Sarcopenic, BMI > 25/Non-Sarcopenic, and BMI > 25/Sarcopenic. The outcomes measured were eGFR (1 and 3 months; and 1, 3, and 5 years), delayed graft function (DGF), rejection, major adverse cardiovascular events (MACE), and post-operative complications. Results: Sarcopenia was associated with an increased 1-year risk of MACE (OR 3.41, *p* = 0.036). BMI alone had no effect on function, DGF, MACE, or on other complications. High VSR was associated with a lower risk of DGF (OR 0.473, *p* = 0.016). When sarcopenia and BMI were assessed together, the BMI > 25/sarcopenic patients had the poorest outcomes, with increased risk of MACE (OR 26.06, *p* = 0.001); poorer eGFR at 1, 3, 12, and 36 months; (*p* < 0.05 at all timepoints), and poorer graft survival (*p* = 0.002). Conclusions: Sarcopenia alone is associated with an increased risk of MACE. Overweight sarcopenic patients are additionally at increased risk of graft loss and have poorer graft function for up to three years.

## 1. Introduction

Sarcopenia is the progressive loss of muscle mass and strength or function, and it is associated with adverse outcomes in various clinical settings, including cancer, chronic liver disease, and end-stage renal disease [1,2,3,4,5]. A particular challenge is the variety of definitions and criteria used in the diagnosis of sarcopenia [6,7,8,9]. Most definitions of sarcopenia include a combination of low muscle mass and strength or physical performance. Usually, the primary step in diagnosis is functional, and it is measured by reduction in muscle strength. As functional limitations may be secondary to other causes, muscle mass assessment is often performed to confirm the diagnosis of sarcopenia. The variation in the definitions of sarcopenia is reflected in the current methods used to measure this entity, including bio-impedance analysis (BIA), dual X-ray absorptiometry (DEXA), handgrip strength, or cross sectional imaging.

While there is no clear evidence in the superiority of any method over another in measuring muscle mass, cross sectional imaging, such as CT scanning, can provide more in-depth, detailed information on body composition. It can be used to assess the mass and volume of skeletal muscle, subcutaneous fat, and visceral fat, as well as the physical interaction through measuring myosteatosis, which has been shown to have functional implications [10,11]. Validated CT measurements used to assess sarcopenia include the skeletal muscle index or psoas muscle index (where the muscle area is adjusted for height), total psoas or abdominal muscle area, and the intramuscular adipose tissue content. Other measures of body composition, such as visceral and subcutaneous fat cross sectional areas, or the ratio of visceral to subcutaneous fat (VSR), can be measured on cross sectional imaging. CT scans can also provide further information beyond body composition and can be used in the pre-operative assessment of patient anatomy, surgical planning, and assessments of vascular calcification.

A more commonly used method of assessing body composition is the body mass index (BMI). Whilst it is not as accurate as other tools, it is simple, easily reproducible, and can be measured in any setting. While BMI is often used to assess nutritional status and sarcopenia, it does not always directly correlate or identify sarcopenic patients in all populations [12,13,14]. Patients with end-stage renal failure on dialysis are a group who may have significant weight fluctuations based on their dialysis schedule. When assessing these patients’ suitability for renal transplantation, BMI is often used as a surrogate marker of body composition and obesity. BMI and obesity are also important clinical parameters post-transplant, with obesity associated with an increased risk of delayed graft function and poorer graft survival [15,16].

Sarcopenia is associated with poor outcomes across a wide variety of populations, including patients undergoing major surgical procedures [5,17,18,19,20]. In transplantation, sarcopenia has been reported mainly in the liver transplant population and is associated with increased rates of graft failure and mortality [21]. In the renal transplant population, the limited evidence suggests that sarcopenia is associated with an increased risk of mortality, post-operative complications, and graft loss in older recipients [22]. Renal failure patients are already a high-risk cohort with multiple comorbidities and sarcopenia may increase this risk further [23].

The aim of our study was to determine the effect sarcopenia has on renal transplant outcomes, not only in isolation, but also as a composite factor with BMI. In addition, we also assessed the interaction of visceral and subcutaneous fat distribution in these patients.

## 2. Materials and Methods

### 2.1. Patient Information

A single-centre retrospective analysis of kidney-only transplants between 2012–2016 were included in this study. All patients with an abdominal CT scan within three years of transplantation, with a follow-up period of 5 years, were included. In our centre, routine pre- or post-operative contrast CT scans are not performed for all transplant patients. All scans were therefore driven by clinical indication, although not necessarily assessments of suspected transplant complications. After screening, 274 out of a total of 497 patients were included in the final analysis. A total of 223 patients were excluded that had not had a CT scan within 3 years or had poor quality imaging preventing accurate measurements of body composition. This study was approved by the local clinical effectiveness unit (approval ID 13916).

Immunosuppression for all patients was in accordance with local protocols. Basiliximab and methylprednisolone were used for induction immunosuppression in low risk patients, and high-risk patients were given anti-thymocyte globulin and methylprednisolone. Maintenance immunosuppression was achieved through a combination of tacrolimus, mycophenolate mofetil, and prednisolone. Post-transplant, aspirin and atorvastatin were prescribed for secondary cardiovascular protection. Antihypertensives, with the exception of beta-blockers, were stopped and reintroduced post-transplant if necessary. Medication including phosphate binders, iron, quinine, and water soluble vitamins prescribed routinely for dialysis patients were stopped. Diabetic patients were routinely reviewed by a diabetes specialist nurse pre-discharge, and all patients were also routinely reviewed by a dietician post-transplant.

### 2.2. Image Analysis

**Figure 1:**
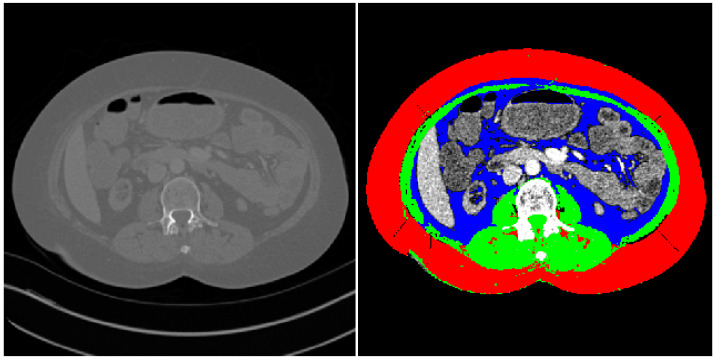
A CT slice at the level of L3 used for assessment of sarcopenia. Points around the peritoneum were traced to enable segmentation of the imported CT slice (**left**) into the final image with a separating of skeletal muscle and visceral, subcutaneous fat (**right**). Red = subcutaneous fat, blue = visceral fat, and green = skeletal muscle.

Sarcopenia was assessed using the semi-automated method previously described by Kim et al. [24]. Measurements were performed by two board-certified radiologists. The CT slice corresponding to the midpoint of the L3 vertebrae was identified and imported into the measurement application. The peritoneum was traced on these reference images to automatically identify skeletal muscle, visceral fat, and subcutaneous fat. All automated image analyses were prospectively assessed for accuracy to rule out miscalculations. Attenuation values of −29 to 150 Hounsfield units were assigned to identify muscle tissue (Figure 1).

### 2.3. Parameters Analysed

The skeletal muscle index (SMI, skeletal muscle area/height^2^) was then calculated based on patient height and the cross sectional skeletal muscle area. Sex-specific cutoffs for SMI were used, with a value of 40.31 for males and 30.88 for females [25]. The BMI at the time of transplant was also measured. Patient outcomes were also analysed based on BMI alone (cutoff 25 kg/m^2^) and by dividing patients into four groups based on a composite of sarcopenia and BMI (BMI < 25/Non-Sarcopenic, BMI < 25/Sarcopenic, BMI > 25/Non-Sarcopenic, and BMI > 25/Sarcopenic).

The visceral/subcutaneous fat ratio (VSR) was measured and analysed using both population-based, sex-specific cutoffs based on the mean VSR (0.628 in females, 1.168 in males) and an alternative cutoff used in the literature (0.5 in females, 1.0 in males) [26].

### 2.4. Outcomes Measured

The outcomes measured were graft function (eGFR, calculated using the CKD-EPI equation [27]) at 1 month, 3 months, 1 year, 3 years, and 5 years. DGF (defined as the need for dialysis within 7 days of transplantation [28]); one month post-operative complications (defined as any medical or surgical deviation from the normal expected post-operative course, including, but not limited to, collections/haematoma, re-exploration, rejection, or urological complications); and one-year major adverse cardiovascular events (MACE) were analysed, as well as graft and patient survival. We also compared episodes of rejection between high- and low-VSR groups.

### 2.5. Statistical Analysis

Statistical analyses was performed using SPSS (version 28, IBM Corp., Armonk, NY, USA). Continuous variables were compared using a t-test or Mann–Whitney U test. Categorical variables were compared using the χ^2^ test. Univariate analyses were performed and factors found to have a *p*-value < 0.10, were subsequently included in the multivariate analyses using general linear models and binary logistic regression. The log-rank test was used to compare graft and patient survival, and *p*-values less than 0.05 were considered statistically significant.

## 3. Results

There were 274 patients included in this study, with 20 patients (7.3%) being sarcopenic. The median SMI in males was 51.3 and 43.9 in females. The mean time between CT scans and transplant was 9.6 months. There were differences in sarcopenic and non-sarcopenic patients’ baseline characteristics, including sex, hypertension, cause of renal failure, and BMI (Table 1).

### 3.1. Sarcopenia-Based Outcomes

Sarcopenia was associated with a significant increase in the risk of 1-year MACE (OR 3.41, *p* = 0.036), but there was no difference in graft function at any timepoint, in the DGF rates, or in other 30-day post-operative complications (Table 2 and Table 3). There was no difference in graft (18.5 vs. 15.6 years, *p* = 0.262) or patient survival (19.4 vs. 17.3 years, *p* = 0.387).

### 3.2. Outcomes Based on BMI

In our study, 124 (45%) patients had a BMI < 25 and 150 (55%) had a BMI > 25. Based on BMI alone, there was no difference in the incidence of MACE (*p* = 0.386), DGF (*p* = 0.252), or 30-day post-operative complications (*p* = 0.267) (Table 4). Mean eGFR was not different at any timepoint (Table 5). There was also no difference in graft (*p* = 0.243) or patient survival (*p* = 0.783).

### 3.3. Outcomes Based on Composite BMI and Sarcopenia

A further subgroup analysis was performed, dividing patients into four groups based on sarcopenia and BMI status (1: BMI < 25/Non-Sarcopenic, 2: BMI < 25/Sarcopenic, 3: BMI > 25/Non-Sarcopenic, and 4: BMI > 25/Sarcopenic). There were 109 patients in Group 1, 15 in Group 2, 145 in Group 3, and 5 in Group 4. There were some differences in the cause of renal failure between each group (*p* = 0.02, Table A1). When assessed as a composite factor, overweight sarcopenic patients (group 4) performed worse compared to the other groups, with an increase in the rates of MACE at 1 year (OR 26.06, *p* = 0.001). When Group 4 was compared individually against each of the other groups, post-transplant eGFR was significantly lower at all timepoints between 1 month and 3 years (*p* < 0.05, Table A2). However, this was not associated with increased rates of DGF or other complications (Table 6).

There were no differences seen in the patient survival across all groups. Overall patient survival was 88.7%, with patient survival rates in Group 4 being worse (80%) but without reaching statistical significance (*p* = 0.198, Table 7). Graft survival was worst in Group 4, with only 40% of grafts surviving at 5 years compared to 72% in the other groups (*p* = 0.029, Table 8).

### 3.4. Outcomes Based on the Visceral-to-Subcutaneous-Fat Ratio

The mean visceral-to-subcutaneous-fat ratio (VSR) in females was 0.628 and 1.168 in males, and these were used as a cutoff in each group. Using these cutoffs, 156 patients had a high VSR, and 118 patients had a low VSR. There was a significant difference in the age and incidence of hypertension in patients with low vs. high VSR (Table A3). Post-operatively, patients with a high VSR were at a lower risk of DGF (OR 0.473, *p* = 0.016). There was no significant difference seen in other post-operative complications or in the eGFR at any timepoint (Table A4 and Table A5). There was also no difference in the overall risk of graft loss or death (*p* = 0.491 and *p* = 0.485, respectively).

A separate analysis was also performed using cutoffs derived from larger published studies [26]. Using a cutoff VSR of 0.5 for females and 1.0 for males, 123 patients had a high VSR, and 151 patients had a low VSR. There was no difference in the eGFR at any timepoint. There was also no difference in the risk of MACE (*p* = 0.341), DGF (*p* = 0.176), rejection (*p* = 0.175), or other post-operative complications (*p* = 0.288) between the groups (Table A6 and Table A7). There was also no difference in patient (20.9 years vs. 20.1 years, *p* = 0.09) or graft survival (19.0 years vs. 17.67 years, *p* = 0.08).

## 4. Discussion

We showed that, in our cohort, sarcopenia alone is significantly associated with cardiovascular outcomes (Table 2). BMI was found to not have a significant effect on the eGFR or on the incidence of MACE, nor on graft or patient survival (Table 4 and Table 5). When combined with high BMI, sarcopenia was not only shown to be a significant risk for MACE, but also for graft function and survival (Table 6 and Table 8).

There have been a small number of studies performed in kidney transplantation that have shown an increased risk of morbidity and mortality in sarcopenic patients post-transplant [22,29,30,31]. However, sarcopenia does not appear to be associated with one-month or one-year major surgical complications [32]. A recent meta-analysis found sarcopenia to be related to poorer quality of life and physical activity, but not to rejection, infection, DGF or death [33]. This meta-analysis was, however, limited by the small number of studies included and high levels of heterogeneity. Despite the lack of large-cohort studies or randomized trials, there is evidence to support the negative impact of sarcopenia on kidney transplant recipients. Our study demonstrates similar results, with no significant effect of sarcopenia on outcomes including post-operative complications, DGF, and all-cause mortality, but it does demonstrate a significantly increased risk of MACE.

Sarcopenia is more commonly associated with the elderly population. However, there is a relatively high prevalence of sarcopenia within the transplant population, affecting an estimated 26% of transplant recipients [33]. One study focusing on sarcopenia in an older transplant population (>60 years) showed an increase in the post-operative length of stay, the total duration of inpatient days during the first year post-transplant, and wound complications in men with low skeletal muscle index [22]. Sarcopenia was also found to be a significant risk factor for the composite endpoint of graft loss or death, with cardiac aetiology being a significant and common cause of death. Whilst our cohort of transplant recipients was much younger (mean age 48.7 years) and as we did not assess length of stay, the increase in MACE in our cohort of sarcopenic and sarcopenic overweight patients highlights the increased cardiovascular risk associated with sarcopenia post-transplant regardless of age. Kim et al. showed a low SMI to be an independent risk factor for post-transplant overall mortality, hospital readmission, and higher creatinine-based eGFR. This is in contrast to our patient survival results, but their study used different cutoff definitions for sarcopenia, and the donor populations were very different between these two studies (65.3% deceased donors compared to 29.3% in the Kim et al. study) [29].

Our results showed that higher VSR is associated with lower risk of DGF; however, there is very limited published literature on the relationship between VSR and transplant outcomes. While increased VSR may be a risk factor for complications in other populations such as patients with gastrointestinal cancers, this effect might be blunted by the improved outcomes associated with having a functioning allograft in the transplant population [34,35]. Larger studies in the transplant population are needed to better understand this effect.

Despite the strong correlation of sarcopenia with patient outcomes in other surgical populations, it has failed to become a part of routine kidney transplant clinical assessments. This may be, in part, due to lack of consensus on the best technique and criteria that should be used for sarcopenia assessment (anthropometric measurements, bioelectrical impedance, DEXA, or cross sectional imaging such an MRI or CT) [36]. Methods such as BMI have remained popular due to their simplicity, but they lack the ability to distinguish between muscle mass and fat and can be misleading, and sarcopenia may be a much more useful prognostic marker than BMI alone [37,38].

Our work is one of the first studies assessing sarcopenia in combination with the routinely used measure of BMI in listing potential recipients. However, we acknowledge there are several limitations to our work. Due to the retrospective nature of this study, functional assessments of sarcopenia and other lifestyle modifications or characteristics, which can affect quality of life, could not be measured. The sarcopenia in this study was assessed only radiologically, which is known to correlate with functional assessments [39,40].

Another significant limitation is the lack of consensus cutoff values for both sarcopenia and VSR, and the low proportion of patients identified as sarcopenic based on the cutoffs used likely underestimated the true numbers of sarcopenic renal transplant patients. The method of assessment and definitions used will have a significant impact on the proportion of patients within any population identified as sarcopenic. In our study, using cutoffs identified from the literature, only 7.3% of our population were sarcopenic, likely identifying patients who were severely sarcopenic. This estimate is on the lower end of the spectrum, with other studies that have assessed sarcopenia in the kidney transplant population reporting the prevalence of sarcopenia at between 4 and 72%, and one meta-analysis even estimated the prevalence at 26% [29,33,41,42]. The development of validated population-specific cutoffs using each method is likely needed to accurately identify sarcopenic-potential-transplant recipients and for the development of intervention strategies to prevent or potentially reverse the process. We experimented with using a different population-specific skeletal muscle index and VSR cutoffs in addition to the ones used in the published literature [43], but we were unable to identify a highly sensitive and specific point to recommend as an appropriate threshold; thus, further studies with a larger population size are required.

The low proportion of sarcopenic patients identified in our study resulted in only five patients in Group 4 (sarcopenic overweight) when subgroup analysis was performed, and these results must be interpreted with caution. There are conflicting results on the impact of BMI alone in post-transplant outcomes, and Schold et al. have suggested BMI may have different implications for different patient groups [15,44,45]. The results of our study may suggest that the sarcopenic cohort is one group where a high BMI is particularly relevant, which results in a significantly increased post-operative risk in sarcopenic overweight patients.

While scans performed within 3 years of transplant potentially includes patients whose body composition and skeletal muscle index may have changed during the study period, we selected CT scans closest to the time of transplant for analysis. The mean time between CT scans and transplant was 9.6 months, an interval that is less likely to lead to significant changes in body composition [46,47,48]. In addition, as not all patients being assessed for transplants have CT scans performed, this study probably reflects a population of more complex and high-risk patients. While this may lead to selection bias, we believe that our analysis becomes more impactful as these high-risk patients are the very cohort where the identification of these newer assessment methods (sarcopenia and VSR) will help clinicians to have a more evidence-based risk stratifications of high-risk recipients.

Finally, interventions to treat or reverse sarcopenia may be beneficial, particularly in this high-risk cohort of patients; hence, identifying these patients early can be useful as aerobic exercise and strength training have been used in the haemodialysis population to improve muscle strength and function with positive results [49]. Routine sarcopenia assessment in a high-risk population can therefore identify patients who will benefit from prehabilitation.

## 5. Conclusions

Our study shows that sarcopenia, but not BMI, in higher-risk kidney transplant recipients is associated with adverse post-operative cardiovascular outcomes. Patients who are sarcopenic and overweight, in addition to the increased cardiovascular risk, also have an increased risk of graft loss and have poorer graft function for up to three years. While CT scans to assess sarcopenia are not routinely performed as part of pre-transplantation assessment, they are often used for other reasons and additional assessments of sarcopenia and body composition may be useful in risk stratification and in the selection of transplant candidates above and beyond the simple BMI assessment. Further studies to assess strategies to target sarcopenia in the transplant population are warranted and may lead to improved short- and long-term outcomes.

## Figures and Tables

**Table 1:** Patient demographics.

	Non-Sarcopenic (*n* = 254)	Sarcopenic (*n* = 20)	*p*-Value
Age (SD)	48.6 (11.9)	50.6 (15.4)	0.479
Sex (% Male)	54.7	90	0.002
Diabetes (%)	26	10	0.111
Hypertension (%)	73.6	50	0.024
Heart disease (%)	18.1	20	0.833
Cause of renal failure (% hypertension/diabetes/ADPKD/IgA nephropathy/others)	10/16/20/10/43	5/10/35/25/25	0.085
Hypercholesterolaemia (%)	37.8	30	0.487
RRT (% pre-emptive/PD/HD)	9.8/18.9/71.3	5.0/15.0/80	0.668
Mean BMI (kg/m^2^)	26.4	22.4	<0.001
Months of RRT pre-transplant (median)	31	27.5	0.910
Transplant type (% LD/DBD/DCD)	35.8/44.5/19.7	20.0/65.0/15.0	0.197

RRT: renal replacement therapy, LD: living donor, DBD: donation after brainstem death, and DCD: donation after circulatory death.

**Table 2:** Multivariate analysis of the post-operative outcomes (grouped by sarcopenia status).

		OR	95% CI	*p*-Value
	MACE			
Non-Sarcopenic		-		
Sarcopenic		3.41	1.082–10.747	0.036
	DGF			
Non-Sarcopenic		-		
Sarcopenic		0.511	0.191–1.366	0.181
	Post-Op Complications			
Non-Sarcopenic		-		
Sarcopenic		1.633	0.645–4.29	0.293

*n* = 20 sarcopenic, 254 non-sarcopenic.

**Table 3:** Multivariate analysis of the post-transplant eGFR in sarcopenic vs. non-sarcopenic patients.

TIME	eGFR (mL/min/1.73 m^2^)		*p*-Value
	Non-Sarcopenic	Sarcopenic	
1 m	50.96	42.43	0.180
3 m	52.24	44.21	0.210
1 y	52.31	49.08	0.620
3 y	48.40	48.03	0.961
5 y	45.91	48.82	0.739

*n* = 20 sarcopenic, 254 non-sarcopenic.

**Table 4:** Multivariate analysis of the post-operative outcomes in the BMI > 25 vs. BMI < 25 patients.

		OR	95% CI	*p*-Value
	MACE			
BMI < 25		-		
BMI > 25		1.325	0.59–2.977	0.386
	DGF			
BMI < 25		-		
BMI > 25		1.378	0.796–2.380	0.252
	Post-Op Complications			
BMI < 25		-		
BMI > 25		1.359	0.791–2.355	0.267

*n*= 124 BMI < 25, 150 BMI > 25.

**Table 5:** Multivariate analysis of post-transplant eGFR in the BMI > 25 vs. BMI < 25 patients.

TIME	eGFR (mL/min/1.73 m^2^)		*p*-Value
	BMI < 25	BMI > 25	
1 m	52.58	48.78	0.209
3 m	53.71	50.20	0.253
1 y	53.74	50.61	0.315
3 y	49.37	47.42	0.540
5 y	47.11	45.03	0.567

*n* = 124 BMI < 25, 150 BMI > 25.

**Table 6:** Multivariate analysis of the post-operative outcomes using sarcopenia and BMI as a composite measure.

		OR	95% CI	*p*-Value
	MACE			
BMI < 25/Non-Sarcopenic		-		
BMI < 25/Sarcopenic		1.395	0.58–7.55	0.699
BMI > 25/Non-Sarcopenic		1.194	0.498–2.862	0.692
BMI > 25/Sarcopenic		26.06	3.566–190.425	0.001
	DGF			
BMI < 25/Non-Sarcopenic		-		
BMI < 25/Sarcopenic		1.222	0.367–4.071	0.744
BMI > 25/Non-Sarcopenic		0.729	0.408–1.303	0.286
BMI > 25/Sarcopenic		4.35	0.453–410.87	0.203
	Post-op complications			
BMI < 25/Non-Sarcopenic				
BMI < 25/Sarcopenic		1.61	0.499–5.199	0.426
BMI > 25/Non-Sarcopenic		1.473	0.829–2.616	0.187
BMI > 25/Sarcopenic		3.947	0.613–25.04	0.148

The *p* values represent each group compared to the BMI < 25/Non-Sarcopenic group, who were used as a baseline for comparison. *n* = 109 BMI < 25/Non-Sarcopenic, 15 BMI < 25/Sarcopenic, 145 BMI > 25/Non-Sarcopenic, and 5 BMI > 15/Sarcopenic.

**Table 7:** Patient survival.

	Patients	Deaths	Survival	*p*-Value
BMI < 25/Non-Sarcopenic	109	11	89.9%	-
BMI < 25/Sarcopenic	15	2	86.7%	0.621
BMI > 25/Non-Sarcopenic	145	17	88.3%	0.705
BMI > 25/Sarcopenic	5	1	80.0%	0.198
Overall	274	31	88.7%	0.604

The overall *p*-value refers to a comparison of all groups using a pooled test. The *p*-values for individual groups refer to pairwise comparisons to the baseline reference group (BMI < 25/Non-Sarcopenic).

**Table 8:** Graft survival rates.

	Patients	Graft Failure	Survival	*p*-Value
BMI < 25/Non-Sarcopenic	109	26	76.1%	-
BMI < 25/Sarcopenic	15	4	73.3%	0.683
BMI > 25/Non-Sarcopenic	145	46	68.3%	0.191
BMI > 25/Sarcopenic	5	3	40%	0.002
Overall	274	79	71.2%	0.029

The overall *p*-value refers to a comparison of all groups using a pooled test. The *p*-values for individual groups refer to pairwise comparisons to the baseline reference group (BMI < 25/Non-Sarcopenic).

## Data Availability

The raw data supporting the conclusions of this article will be made available by the authors on request.

## Appendix A

**Table A1:** Causes of renal failure in each patient using BMI and sarcopenia as a composite measure.

Cause of Renal Failure	BMI < 25/ Non-Sarcopenic	BMI < 25/ Sarcopenic	BMI > 25/ Non-Sarcopenic	BMI > 25/ Sarcopenic	*p*-Value
Hypertension (%)	9.1	6.7	11.7	0	0.74
Diabetes (%)	11.9	0	19.3	40	0.05
ADPKD (%)	15.6	40	23.4	20	0.13
IgA nephropathy (%)	10.1	20	11.0	40	0.16
Other (%)	53.2	33.3	34.5	0	0.04

*n* = 109 BMI < 25/Non-Sarcopenic, 15 BMI < 25/Sarcopenic, 145 BMI > 25/Non-Sarcopenic, and 5 BMI > 25/Sarcopenic. Overall *p*-value = 0.02.

**Table A2:** Multivariate analysis of post-transplant eGFR using BMI and sarcopenia as a composite measure.

Time	eGFR (mL/min/1.73 m^2^)			
	BMI < 25/Non-Sarcopenic	BMI < 25/Sarcopenic	BMI > 25/Non-Sarcopenic	BMI > 25/Sarcopenic
1 m	52.37, * *p* = 0.002	52.24, * *p* = 0.007	49.56, * *p* = 0.004	14.72
3 m	53.79, * *p* = 0.016	51.35, * *p* = 0.052	50.83, * *p* = 0.029	24.09
1 y	53.44, * *p* = 0.038	55.65, * *p* = 0.045	50.80, * *p* = 0.059	24.54
3 y	49.44, * *p* = 0.011	49.39, * *p* = 0.027	47.17, * *p* = 0.016	15.55
5 y	46.22, * *p* = 0.411	48.74, * *p* = 0.788	43.09, * *p* = 0.540	8.0

* *p*-values represent pairwise comparisons with Group 4 (BMI >25/Sarcopenic) patients. *n* = 109 BMI < 25/Non-Sarcopenic, 15 BMI < 25/Sarcopenic, 145 BMI > 25/Non-Sarcopenic, and 5 BMI > 25/sarcopenic.

**Table A3:** Patient demographics based on the visceral-to-subcutaneous-fat ratio.

	High VSR	Low VSR	*p*-Value
Age (SD)	51.61 (10.34)	46.68 (13.03)	0.001
Sex (% Male)	57.0	57.5	0.937
Diabetes (%)	25.4	24.3	0.841
Hypertension (%)	78.9	66.3	0.014
Heart disease (%)	21.9	15.7	0.183
Cause of renal disease (% Hypertension/diabetes/ADPKD/IgA nephropa-thy/others)	12.3/13.2/23.7/11.4/39.4	8.8/17.5/19.4/11.9/42.5	0.124
Hypercholesterolaemia (%)	42.1	33.8	0.158
RRT (% pre-emptive/PD/HD)	10/17.5/72.5	8.8/20.2/71	0.826
Mean BMI (kg/m^2^)	26.7	25.7	0.09
Months of RRT pre-transplant (median)	37.2	37.3	0.98
Transplant type (% LD/DBD/DCD)	37.7/43.9/18.4	32.5/46.3/20	0.714

VSR: visceral-to-subcutaneous-fat ratio. VSR cutoffs: males 1.16, females 0.63. *n* = 156 high VSR, 118 low VSR.

**Table A4:** Multivariate analysis of the post-transplant eGFR in high- vs. low-VSR patients.

Time	eGFR (mL/min/1.73 m^2^)		*p*-Value
	High VSR (*n* = 156)	Low VSR (*n* = 118)	
1 m	50.86	50.12	0.808
3 m	51.27	52.12	0.784
1 y	50.96	52.80	0.558
3 y	49.15	47.45	0.600
5 y	44.31	45.07	0.840

VSR: visceral-to-subcutaneous-fat ratio. VSR cutoffs: males 1.16, females 0.63.

**Table A5:** Multivariate analysis of the post-transplant outcomes in high- vs. low-VSR patients.

Group		OR	95% CI	*p*-Value
	MACE			
Low VSR		-		
High VSR		1.335	0.622–2.866	0.458
	DGF			
Low VSR		-		
High VSR		0.473	0.257–0.868	0.016
	Rejection			
Low VSR		-		
High VSR		0.618	0.235–0.623	0.329
	Post-op Complications			
Low VSR		-		
High VSR		0.965	0.582–1.600	0.889

VSR: visceral-to-subcutaneous-fat ratio. VSR cutoffs: males 1.16, females 0.63. *n* = 156 high VSR, 118 low VSR.

**Table A6:** Multivariate analysis of the post-transplant eGFR in high- vs. low-VSR patients (alternative cutoffs).

Time	eGFR (mL/min/1.73 m^2^)		*p*-Value
	High VSR	Low VSR	
1 m	48.7	52.9	0.173
3 m	50.1	54.1	0.202
1 y	50.7	53.7	0.158
3 y	47.3	49.6	0.472
5 y	43.1	46.9	0.226

VSR: visceral-to-subcutaneous-fat ratio. VSR cutoffs: males 1.0, females 0.5. N = 123 high VSR, 151 low VSR.

**Table A7:** Multivariate analysis of the post-transplant outcomes in high- vs. low-VSR patients (alternative cutoffs).

Group		OR	95% CI	*p*-Value
	MACE			
Low VSR		-		
High VSR		1.476	0.663–3.288	0.341
	DGF			
Low VSR		-		
High VSR		0.670	0.376–1.196	0.176
	Rejection			
Low VSR		-		
High VSR		0.523	0.205–1.334	0.175
	Post-op Complications			
Low VSR		-		
High VSR		1.316	0.793–2.186	0.288

VSR: visceral-to-subcutaneous-fat ratio. VSR cutoffs: males 1.0, females 0.5. *n* = 123 high VSR, 151 low VSR.
